# Mapping the mutational landscape of an avian retrovirus envelope protein across its evolutionary trajectory

**DOI:** 10.1371/journal.ppat.1014110

**Published:** 2026-03-31

**Authors:** Moru Xu, Yupeng Liu, Xin Lan, Zhenyu Wang, Kun Qian, Hongxia Shao, Jinlin Huang, Jianqiang Ye, Aijian Qin

**Affiliations:** 1 Ministry of Education Key Lab for Avian Preventive Medicine, College of Veterinary Medicine, Yangzhou University, Yangzhou, Jiangsu, P.R. China; 2 Jiangsu Co-innovation Center for Prevention and Control of Important Animal Infectious Diseases and Zoonoses, Yangzhou, Jiangsu, P.R. China; 3 Key Laboratory of Prevention and Control of Biological Hazard Factors (Animal Origin) for Agri-Food Safety and Quality, Ministry of Agriculture of China, Yangzhou University, Yangzhou, China; Instituto Nacional de Cancer, BRAZIL

## Abstract

Many RNA viruses exhibit error-prone replication. Continuous generation of erroneous copies accelerates evolution. Avian leukosis virus subgroup J (ALV-J), an avian oncogenic virus, is a classical model virus for studying retroviruses. ALV-J's high mutation rates drive continuous evolution of its envelope and pathogenicity, posing significant challenges to the poultry industry. Here we employed deep mutational scanning to systematically assess envelope-wide mutation effects on ALV-J replication, integrating high-throughput sequencing with mutant libraries to identify critical envelope residues impacting viral fitness. Following 10 passages, the library virus exhibited enhanced replication capacity. Moreover, the library virus derived from SPF chickens displays screening results similar to those of the DF-1 cell-passaged virus. Most mutations were progressively eliminated during viral passaging, especially the first 80 amino acids of ALV-J envelope. Critical amino acid mutations, preferential deletion/insertion mutations and glycosylation patterns recapitulate evolutionary patterns observed in natural ALV-J isolates. Incorporation of all identified mutations into ALV-J J1 significantly increased in *vivo* replication efficiency and viral shedding of the recombinant virus. Functional study demonstrated that two key mutations independently promote viral replication: A64T enhancing entry via receptor-binding optimization, H304R promoting maturation through envelope cleavage efficiency. These insights enable targeted antiviral design by predicting evolutionary paths.

## Author summary

RNA viruses frequently undergo error-prone replication, generating genetic variants that accelerate evolutionary adaptation. Avian leukosis virus (ALV), a tumorigenic retrovirus causing immunosuppression in chickens, represents a major economic burden to poultry. As a prime model for viral evolution, subgroup J ALV (ALV-J) displays extraordinary genetic diversity, with Env proteins showing low amino acid conservation due to rapid mutation. These adaptive mutations alter receptor binding, tissue tropism, and host range, presenting major challenges for therapeutic development and enabling persistent infections. To investigate ALV-J evolution, we performed deep mutational scanning of the envelope protein, combining mutant libraries with high-throughput sequencing to identify fitness-critical residues. After 10 passages, we observed enhanced viral replication with progressive elimination of most mutations, particularly in the N-terminal 80 residues. Consistent screening results were observed both in *vivo* or in *vitro*. The evolved ALV-J carrying all selected mutations showed enhanced in *vivo* replication and shedding. Notably, we identified two functionally distinct mutations: A64T improved receptor binding for cell entry, while H304R enhanced envelope cleavage efficiency for viral maturation. These findings mirror natural evolutionary patterns and provide a predictive framework for developing targeted antivirals against ALV-J.

## Introduction

RNA viruses often showed high mutation rates, enabling rapid emergence of new strains with expanded host ranges or geographic spread [1]. The accelerated evolution arises from rapid replication, error-prone RNA polymerase, and frequent recombination. Positive selection often favor mutations in viral surface proteins, resulting in the formation of viral quasispecies with enhanced fitness and adaptability [2], while negative selection limits diversity by removing deleterious mutations [3]. The rapid mutation of viruses poses significant challenges to drug/vaccine/antibody development, driving persistent infections and spread [4,5]. To achieve effective viral control, a critical question must be addressed: what are the evolutionary pathways of the virus?

Avian leukosis/sarcoma virus (ALV/ALSV) is an enveloped, positive sense RNA virus belonging to the family Retroviridae [6]. ALV infection can induce serious tumors in chicken, together with decreased production performance and immune suppression, causing significant economic loss. ALV in chicken is classified into 7 subgroups, with subgroup J (ALV-J) demonstrating exceptional epidemiological significance [7]. ALV-J exhibits remarkable genetic diversity and rapid evolution, with a spontaneous mutation rate of 4.6 × 10^−5^ mutations per site per generation [8]. Phylogenetic analyses reveal that ALV-J has diversified into multiple distinct lineages [9], which show strong associations with host genetic background and geographical distribution [10]. The predominant pandemic strains can be traced to a common ancestor isolated approximately 35 years ago [11]. Descendants of this virus spread globally and underwent rapid evolution, with genetic divergence primarily concentrated in the envelope protein (Env) and the 3’ untranslated region (3'UTR). Most mutations were concentrated in the variable regions (vr2, vr3) and hypervariable regions (hr1, hr2), including amino acid substitutions and insertion/deletion mutations [12–16]. Most N-linked glycosylation site (NGS) within ALV-J were conserved, but different strains either gain or lose some NGSs [17].

The rapid evolution of ALV-J Env alters viral replication kinetics, receptor binding specificity, tissue tropism, oncogenic potential, and host spectrum expansion [18–22]. Since its emergence, ALV-J have successively infected White Leghorn broilers, layer hens [18] and indigenous chickens in China [23]. ALV-J layer strains replicate faster than the prototype strain HPRS103 [21]. ALV-J Env is further divided into SU (gp85) and TM (gp37) subunits. A single mutation at N123I of SU improved viral replication through enhancing the receptor binding affinity with Na+/H+ exchanger type 1 (NHE1) on host cell surface [21]. In addition, different ALV-J strains showed diverse cell and tissue preference. Most of the early ALV-J strains usually caused myelocytomatosis, while since 2004, many layer chicken isolates were shown to induce hemangioma [18]. Recently, many field cases reported hepatomegaly and splenomegaly in ALV-J-affected chickens [24]. Mutation of a critical NGS in ALV-J Env can overcome the cellular resistance and broaden host tropism [22]. Therefore, gaining a comprehensive understanding of the evolutionary steps and the changes in the key amino acid residues of ALV-J Env is of critical importance.

Here, we integrated deep mutational scanning (DMS) with high-throughput sequencing to systematically analyze selective pressures on ALV-J envelope proteins. DMS employs error-prone PCR to generate comprehensive variant libraries encompassing all possible nucleotide substitutions. These mutant libraries are then recombined into viral backbones for functional rescue and subsequent selection. The viral population undergoes specific selection conditions, with deep sequencing subsequently quantifying allele frequency changes [25]. Study on HIV-1 Env used DMS to screen for amino acid residues affecting viral replication capacity [26], epitopes recognized by neutralizing antibodies or T cells [27] and evolutionary landscape among different strains [28]. Complementary techniques including random transposon mutagenesis and comprehensive deletion library sequencing have enabled genome-wide mapping of non-essential genes or viral cis-and trans-acting elements [29,30].

We combined DMS with ALV-J epidemiological data to identify key mutations matching natural strain evolution. By analyzing positive and negative selection results in *vitro* or in *vivo,* and correlating the findings with conservation patterns in natural isolates, we pinpointed evolutionarily critical sites and conserved functional elements. Investigating how these sites modulate Env protein function offers new insights for viral pathogenicity and control strategies.

## Results

### Construction and serial passage of an ALV-J envelope protein mutant library

**Fig 1:**
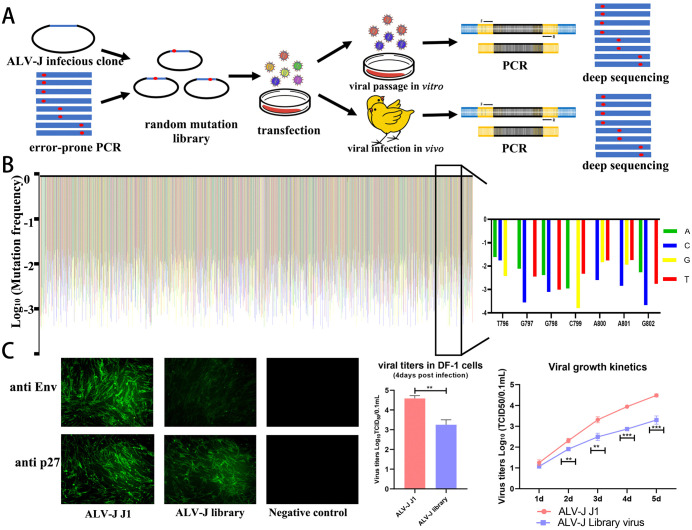
Creation of random mutation library of ALV-J Env. (A) Schematic of mutation library construction. (B) High-throughput sequencing identified 2,437 high-quality mutations. (C) DF-1 cells were transfected with infectious clone of ALV-J J1 or ALV-J mutant library. Immunofluorescence were applied to detect viral p27 and Env protein. Supernatants were collected for replication kinetics.

Error-prone PCR was used to generate ALV-J env fragments containing random mutations. Following recombination, we constructed a full-length envelope mutant library (Fig 1A), theoretically containing 2,772 single-nucleotide mutations across a 924 bp randomized region. Sanger sequencing confirmed a mutation frequency of ~16 mutations/kb. Approximately 20,000 bacterial colonies were collected to ensure >100 × coverage. High-throughput sequencing (Fig 1B) identified 2,437 distinct mutations (87.9% gene coverage) with frequencies from 3 × 10 ⁻ ⁴ to 1.2 × 10 ⁻ ². Post-transfection, the mutant library and wild-type ALV-J J1 exhibited comparable replication levels of p27 protein. However, Env expression was significantly reduced in the mutant library, leading to a marked decrease in viral titer and replication rate (Fig 1C).

**Fig 2:**
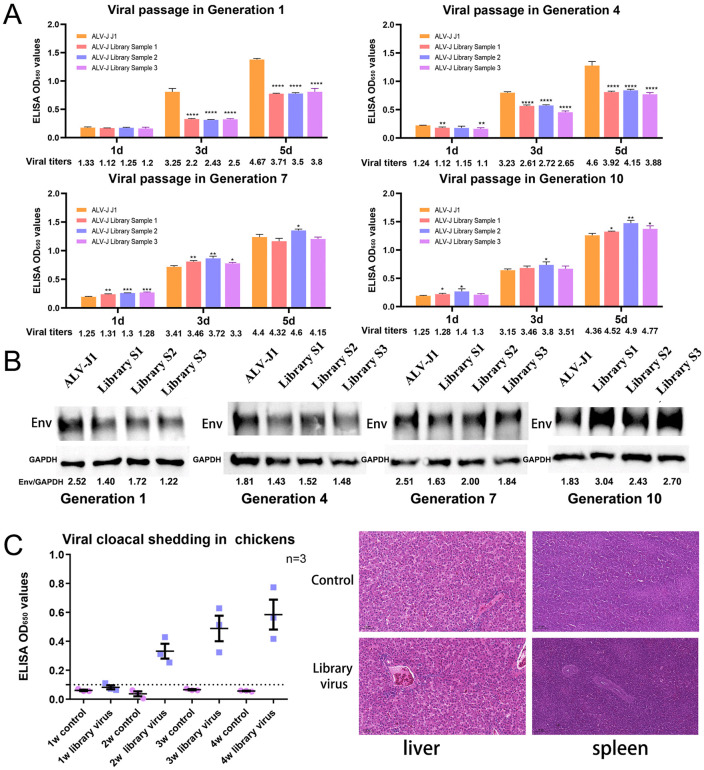
Continuous passage and replication of mutant ALV-J in DF-1 cells. (A) DF-1 cells were inoculated with ALV-J J1 or ALV-J mutant library (MOI = 0.1) for 5 days. Supernatants were collected for viral titer and capsid p27 ELISA across passages 1–10. (B) Env protein expression in DF-1 cells infected with mutant or wild-type ALV-J over 10 generations, with GAPDH being the housekeeper gene. (C) Viral shedding in cloacal swabs from SPF White Leghorns inoculated with the library virus during weeks 1–4 post-inoculation. (D) Three 1-day-old SPF White Leghorns were inoculated with 5,000 TCID₅₀ ALV-J library virus. Histopathological examination of chicken liver and spleen 30 days post-inoculation.

The wild-type ALV-J J1 and the mutant library were passaged for 10 consecutive generations. Viral replication was assessed according to viral titer (S1 Fig), p27 level (ELISA) (Fig 2A) and Env expression (western blot) (Fig 2B). ALV-J J1 maintained a stable replication rate from G1 to G10. In contrast, the three replicate mutant library populations showed attenuated replication at the early generations (G1 and G4). However, their replication efficiency progressively increased. By G10, the mutant viruses outperformed the parental strain.

Chickens inoculated with 5,000 TCID_50_ of the mutant library virus stably shed the virus during weeks 2–4 (Fig 2C). Histopathological examination revealed no detectable lesions in the liver or spleens at 30 days post-inoculation (Fig 2D). However, Antibodies against ALV-J SU and p27 were detected in chickens on days 15 and 30 (S2 Fig), indicating that adaptive immunity exerts selective pressure during viral evolution.

### DMS revealed the dynamic changes of all mutations during viral passaging

**Fig 3:**
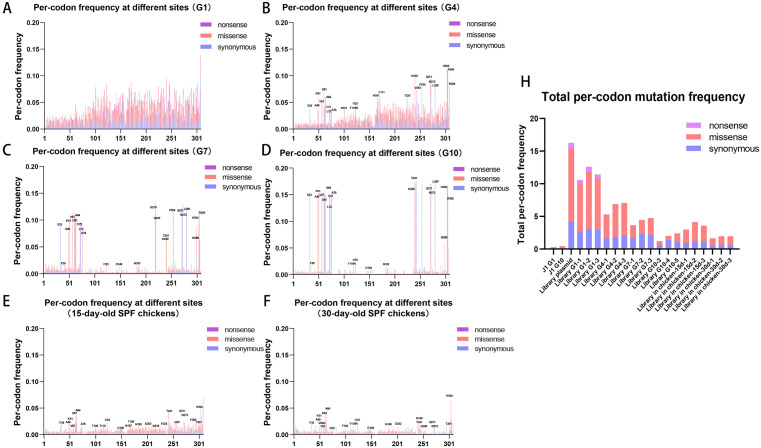
Per-codon mutation frequency of different viruses at all positions. (A-F) Per-codon mutation frequency analysis showing amino acid positions across viral variants, with nonsense (purple), missense (red), and synonymous (blue) mutations annotated. Key mutational hotspots showing preferential patterns are indicated. (G) Total mutation frequency profiles of wild-type ALV-J J1 (virus), plasmid mutant libraries (DNA), derived mutant viruses across generations 1–10 (G1-G10) and those from SPF chicken.

Viral mutations per passage were quantified via Illumina sequencing. Replication Capacity (RC) values were derived from mutation frequencies relative to the plasmid baseline. Pearson correlation analysis (r) across three replicate samples demonstrated moderate repeatability for G1/G4 viral passages (r = 0.35-0.71), with improved repeatability in G7/G10 passages (r = 0.71-0.82). Furthermore, the virus exhibited high repeatability in 30-day-old SPF chickens (r = 0.57-0.80) (S3 Fig). Nonsense mutations, observed only in G1, were likely lethal and were rapidly purged. Missense mutations decreased, suggesting deleterious effects, while synonymous mutations persisted and dominated in later generations. In addition, we observed mutation hotspots emerging from G4 onward, with their proportion gradually increasing during subsequent viral passaging (Fig 3A-D). Interestingly, we found comparable results in SPF chickens (Fig 3E/F). The total viral mutation frequency declined steadily during passaging, dropping to 17% of initial levels by G10 (Fig 3G). The wild-type ALV-J were passaged as a control and showed a low overall mutation frequency.

**Fig 4:**
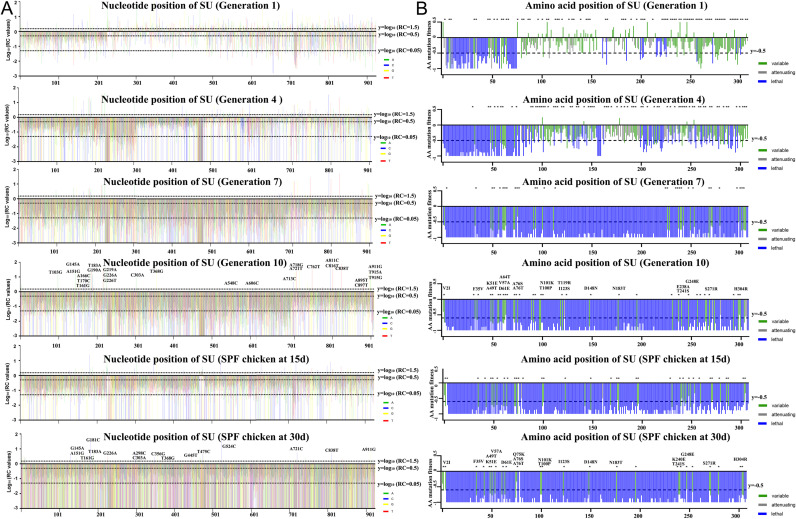
Identification of lethal and preferred mutations using quantitative highthroughput genomics. (A) Log_10_ RC index of all individual point mutations in mutant library virus after 10 generations and from SPF chicken. Reference lines indicate the logarithmic transformations of RC = 1.5, RC = 0.5, and RC = 0.05 for comparison. (B) The amino acid mutation fitness values (AA mutation fitness values) of all point mutations. Based on the calculated values, different amino acid site mutations are classified into three categories: lethal, attenuating and variable. The variable mutations were also marked with ‘*’ on the top.

We calculated the RC value of 2,437 nucleotide mutations in each sample (Fig 4A). Apart from a few mutations with RC values ≤ -3 (including low-confidence sequencing data and those containing stop codons), the majority exhibited a normally distributed clustering pattern (S4 Fig). Mutations were categorized as A (enhancing), B (neutral), C (attenuating), or D (lethal) based on RC values. With successive viral passages, majority of mutations shifted from tiers A/B to tiers C/D, indicating progressive impairing/lethal selection. The number of mutations classified as tier A (RC > 1.5) decreased progressively during viral passages, from 7.9% to 2.7% (S4 Fig). From G4 to G10, we observed a subset of mutations exhibiting high RC values (Fig 4A) (Table 1).

**Table 1:** Key nucleotide substitutions identified from ALV-J mutation library (Generation 10).

No.	Nucleotide substitution	Amino acid substitution	RC values in *vitro* (G10)	RC values in *vivo* (30d)	
1	G4A	V2I	3.00	0.96	
2	T103G	F35V	1.66	2.28	
3	G145A	A49T	24.21	4.03	
4	A151G	K51E	24.36	4.75	
5	T161G	V54G	3.49	4.55	
6	A166C	N56H	3.42	0.04	
7	T170C	V57A	13.32	1.56	
8	T183A	D61E	19.49	3.53	
9	G190A	A64T	18.40	6.05	
9	G219A	L73L	35.81	0.60	
10	C223A	Q75K	0.17	3.27	
11	G226A	A76T	43.59	3.04	
12	G226T	A76S	2.21	3.16	
13	A298C	T100P	1.78	2.31	
14	C303A	N101K	8.82	2.05	
15	A335C	Y112S	1.61	0.81	
16	G349C	G117R	1.63	0.87	
17	C356G	T119R	12.18	5.39	
18	T368G	I123S	1.77	1.54	
19	G442A	D148N	1.54	1.04	
20	C468A	V156V	9.61	2.54	
21	A548C	N183T	1.74	1.21	
22	A606C	E202D	1.71	1.75	
23	A713C	E238A	4.62	0.27	
24	A718G	K240E	23.47	2.25	
25	A721T	T241S	25.37	0.50	
26	A721C	T241P	0.56	6.01	
27	G743A	G248E	0.87	2.38	
28	A811C	S271R	121.07	6.00	
29	C838T	L280L	79.20	3.10	
30	A911G	H304R	21.28	6.26	
31	T915A	R305R	15.71	1.84	
32	A916G	S306G	6.20	0.81	

We sought to characterize the functional consequences of all possible point mutations in ALV-J Env protein. Because a single amino acid change can result from more than ten different nucleotide substitutions, each potentially exerting distinct effects on viral fitness, we implemented an integrated computational framework to systematically evaluate mutational impacts at the protein level, moving beyond analyses of individual nucleotide changes (Fig 4B). Most mutations were eliminated during passaging, with particularly strong negative impacts observed in the first 80 residues (even G1 variants were purged). High-RC mutations are shown in Fig 4B and Table 1.

### DMS reveals preferential deletion/insertion mutations and glycosylation patterns matching natural isolates

**Fig 5:**
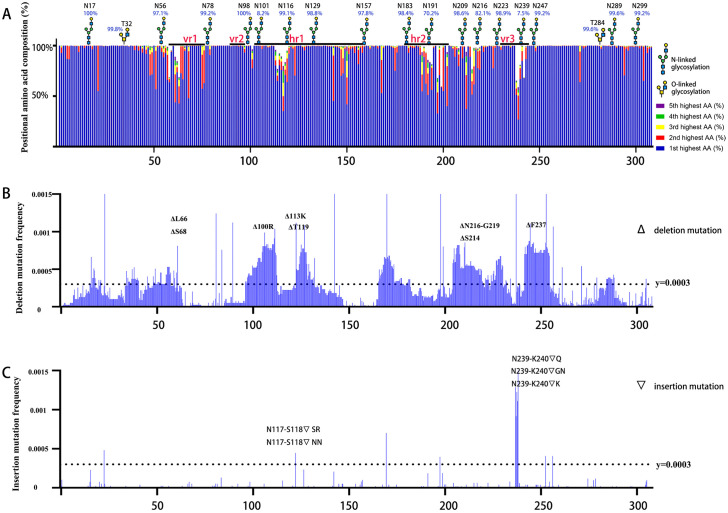
DMS reveals selective preferences for glycosylation sites and insertion/deletion mutation mutations in ALV-J SU. (A) Positional amino acid composition of ALV-J SU among 572 natural isolates. At each position, the top five most abundant amino acids are colored. Variations in column length reflect the presence of indels mutations. The variable regions vr1, vr2 and vr3, hypervariable regions hr1 and hr2 are annotated. N/O-glycosylation sites and their proportions are marked. (B) Deletion mutation profile in the ALV-J mutant library. Deletion mutation frequencies are displayed. In-frame deletions are specifically labeled, showing their corresponding amino acid deletions. (C) Insertion mutation profile in the ALV-J mutant library. Insertion mutation frequencies are displayed. Positions with in-frame insertions that lead to amino acid insertions are highlighted.

We analyzed amino acid composition across all available ALV-J envelope sequences from GenBank (S1 Table), identifying characteristic substitutions, deletions/insertions, and glycosylation site variations (Fig 5A). Mutations clustered at positions 45–75, 110–120, 145–155, 185–220, and 238–245, showing moderate divergence from the traditionally defined variable regions (vr1, vr2 and vr3) and hypervariable regions (hr1, hr2). The glycosylation sites located in the N-and C-terminal regions of the envelope protein are conserved, whereas those in the middle segment exhibit lower conservation, particularly at sites N101, N191, N216, and N239.

DMS revealed distinct patterns in the preference for insertion and deletion mutations. Deletions clustered prominently within regions at amino acids 60–80, 95–125, 170–180, 205–230, and 240–255 (Fig 5B), whereas insertions were less frequent and concentrated at amino acids 239–245 (Fig 5C). The hotspots overlap with genomic regions where natural viral strains frequently display indels. Notably, some mutations introduced amino acid indels identical to those found in natural variants (S2 Table).

DMS data showed dynamic changes of glycosylation sites (S3 Table). During passaging, most glycosylation site mutants were progressively purged. N- and C-terminal variants were eliminated early, whereas central-region mutants persisted longer under selection.

**Fig 6:**
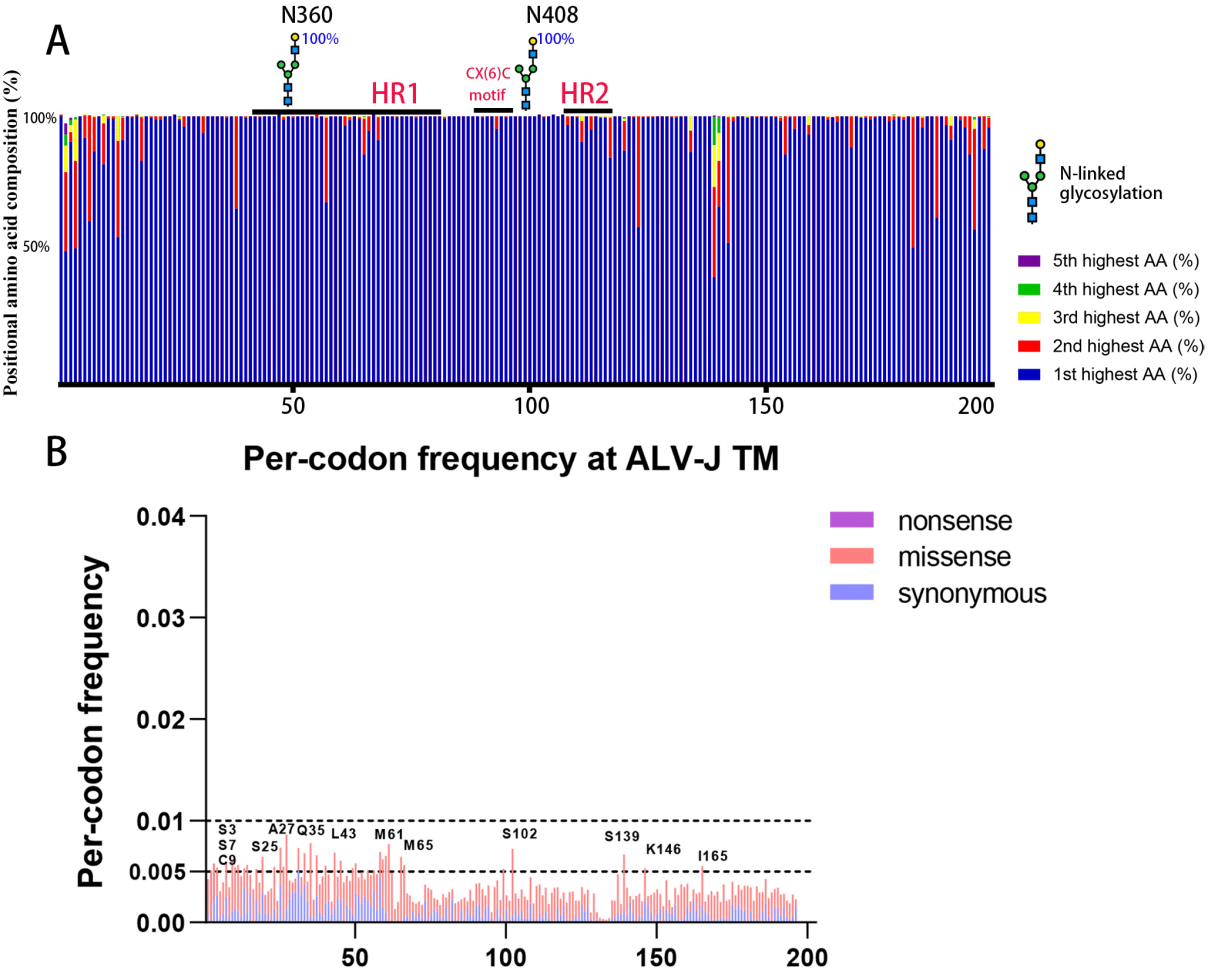
DMS revealed no significant variation in the ALV-J TM. (A) Positional amino acid composition of ALV-J TM among 340 natural isolates. At each position, the top five most abundant amino acids are colored. Variations in column length reflect the presence of indels mutations. Heptad repeat 1 (HR1) and heptad repeat 2 (HR2) are annotated. N-linked glycosylation sites and their proportions are marked. (B) Point mutation profile in the ALV-J mutant library (G10). Frequencies of mutations are plotted across all sites.

ALV-J's TM is highly conserved across all natural strains, with only a few sites exhibiting mutations (Fig 6A). To investigate whether TM provides compensatory mutations for changes within SU, we implemented a dual-sequencing approach (Sanger and high-throughput sequencing) for TM (gp37) analysis. Based on the results of ALV-J library virus in DF-1 cells (G10), more than 10 sites in ALV-J TM exhibited mutation frequencies greater than 0.5%, but none exceeded 1% (Fig 6B).

### DMS identifies critical amino acid mutations consistent with the evolutionary trajectory of natural isolates

**Fig 7:**
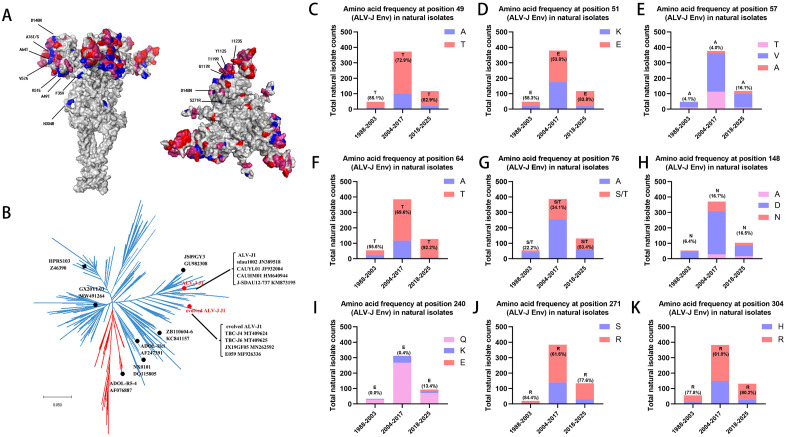
DMS uncovered naturally selected amino acid preferences in the ALV-J Env and a shift in the viral phylogenetic clustering. (A) ALV-J Env trimer surface model (J1 strain) showing conservation levels: ≥ 90% (grey), 60–90% (pink), ≤ 60% (red). Substituted residues are highlighted in blue. (B) Maximum-likelihood phylogenetic tree was generated using MEGA X with 1,000 bootstrap replicates. Two major clades are highlighted in red and blue, respectively. Strains closely related to ALV-J J1 or its evolved mutants were specifically labeled. (C-K) Nine amino acid substitutions were chosen with high RC values. 572 ALV-J strains were categorized into 3 groups according to their isolation time (1988–2003, 2004–2017 and 2018–2025). The proportional changes (top two or three most abundant amino acids) were examined in natural isolate.

DMS revealed 32 evolutionarily significant nucleotide substitutions in ALV-J envelope with high RC values (Table 1). All these sites, with the exception of H304R, are located in the head region of the Env protein trimer (Fig 7A). By integrating absolute mutation frequency thresholds (Fig 3) with Sanger sequencing validation (S5 Fig), we prioritized nine amino acid changes for further analysis. To assess temporal trends, we categorized 592 ALV-J isolates from GenBank into three evolutionary periods: 1988–2003, 2004–2017, and 2018–2025. All nine selected mutations exhibited increasing frequencies across these periods (Fig 7C-K), suggesting potential adaptive advantages in viral fitness.

Following incorporation of the 32 selected nucleotide substitutions into the ALV-J J1 strain, the resulting recombinant virus, designated as ‘evolved ALV-J J1,’ exhibited a significantly altered phylogenetic position. The original ALV-J J1 strain showed the greatest homology to ALV-J isolates from 2009-2012, whereas the ‘evolved ALV-J J1’ exhibited the highest similarity to those from 2017-2019 (Fig 7B).

### The recombinant ALV-J carrying all selected mutations showed enhanced replication and shedding in *vivo*

**Fig 8:**
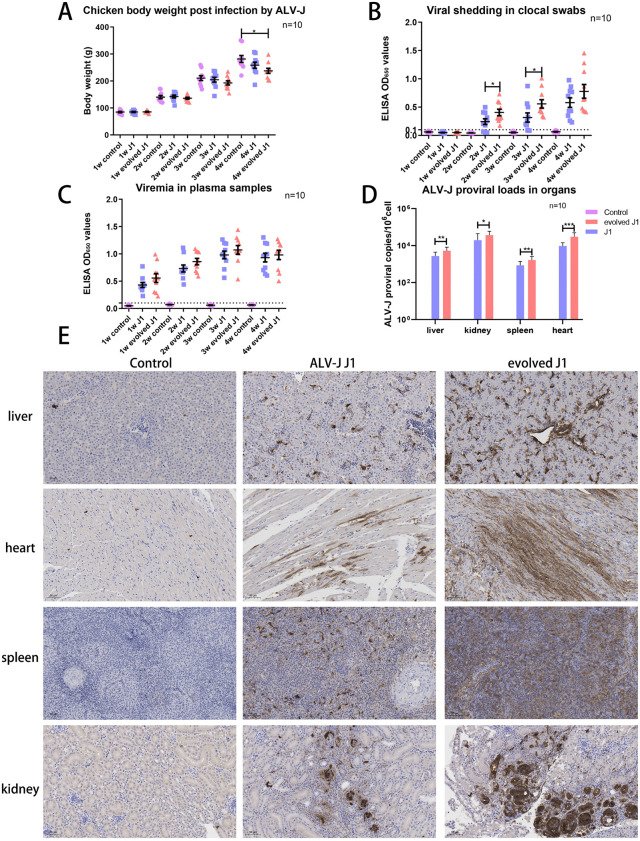
The evolved ALV-J carrying all selected mutations showed enhanced viral replication and shedding in *vivo.* (A) Body weight of chickens in three groups during weeks 1–4. (B) Viral shedding in cloacal swabs from three groups of chickens during weeks 1–4. (C) Viremia levels in three groups of chickens during weeks 1–4. (D) Viral loads in heart, liver, kidney, and spleen of chickens from three groups at day 30. (E) Detection of ALV-J protein in tissues from three groups by immunohistochemistry (IHC).

We inoculated 1-day-old SPF chickens with either 10,000 TCID_50_ of ALV-J strain J1 or the evolved J1, and maintained them for 30 days. ALV-J infection resulted in reduced weight gain, especially seen in the evolved J1 group (Fig 8A). Through ELISA detection of ALV-J p27 protein in cloacal swabs, we found that viral shedding began at week 2 post-infection in both challenged groups. However, chickens in the evolved J1 group exhibited significantly higher viral shedding levels compared to the J1 group (Fig 8B). All chickens inoculated with either J1 or evolved J1 strains exhibited persistent viremia beginning at week 1 post-infection (Fig 8C). Viral loads of liver, kidney, spleen and heart in the evolved J1 groups were 11.4-13.4 times higher than the J1 group (Fig 8D). The immunohistochemical (IHC) results demonstrated that chickens in the evolved J1 group exhibited significantly higher numbers of ALV-J-positive cells in the liver, heart, spleen and kidney tissues compared with the other groups (Figure 8E).

### Mutation of key amino acids results in advantages in viral replication capacity and in Env protein function

**Fig 9:**
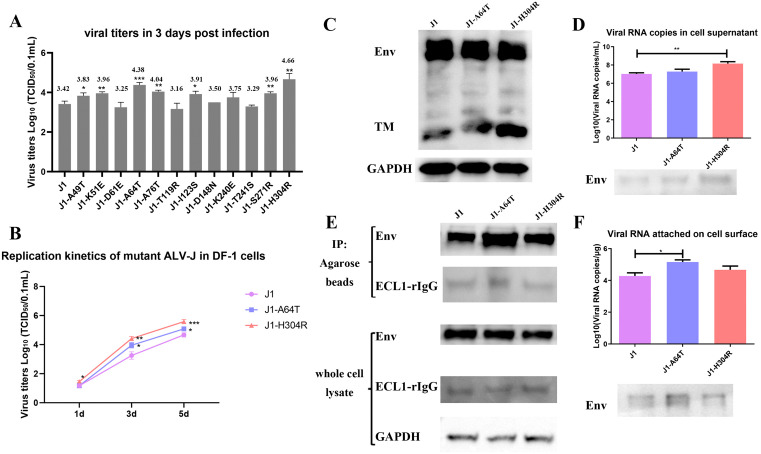
Key amino acid substitutions promote ALV-J replication. (A) Viral titers of single-point mutant ALV-J strains at 3 days post-infection (MOI = 0.1) were determined by TCID_50_ assay. (B) Replication kinetics of wild-type J1 and mutant strains (J1-A64T, J1-H304R) were compared at 1–5 days post-infection (MOI = 0.1) using TCID_50_ titration. (C) Env protein processing was analyzed by western blot. (D) Viral genomic RNA (RT-qPCR) and Env protein (western blot) levels in cell supernatants were quantified. (E) DF-1 cells were co-transfected with plasmid expression of various Env and NHE1-ECL1-rIgG. Co-immunoprecipitation (Co-IP) was performed using anti-Flag antibodies, followed by Western blot to verify protein interaction. (F) Viral attachment was assessed by measuring cell-associated viral RNA (RT-qPCR) and Env protein (western blot) after 2h incubation at 4 °C with equal viral inputs.

Considering the short Illumina reads (150 bp) do not allow us to evaluate the presence of multiple mutations in individual clones, we implemented Sanger sequencing for SU gene analysis. Results showed an average of 2–4 mutations per SU gene (S5 Fig) in G10 virus. We aimed to introduce each mutation individually into the J1 strain to assess the impact of these site-specific mutations on viral properties. To minimize interference from multiple mutations, we selected the 12 most frequently mutated sites for single-point mutagenesis based on results from Table 1 and S5 Fig. Our analysis revealed that mutations at 7 of these sites conferred replication advantages (Fig 9A). Notably, the A64T and H304R mutations demonstrated the most significant enhancement of viral replication. (Fig 9B).

The H304R mutation is located between the SU and TM subunits of ALV-J Env, precisely at the furin protease cleavage site. We suspect that the H304R mutation enhances the cleavage efficiency of the Env, thereby boosting infectious virion production. Western blot showed that H304R yielded similar amounts of Env protein in cell lysates, but higher level of SU (Fig 9C). In addition, J1-H304R showed more Env protein and viral RNA in the cell supernatant, indicating mutation at this site can generate more virions (Fig 9D).

The A64T mutation is located in the Env protein's receptor-binding domain that interacts with NHE1. Mutation of A64T would add an additional N-linked glycosylation site which was seen in 72.7% of natural ALV-J J1 strains. Expression and purification of ALV-J SU from DF-1 cells, followed by mass spectrometry, revealed an N-glycosylation at the N62 site (1.2 kDa) following the A64T mutation (S6 Fig). We aim to investigate whether this mutation enhances viral replication by increasing Env-NHE1 binding affinity. We demonstrated that NHE1-ECL1-rIgG, the first extracellular loop of NHE1 that was fused to rabbit IgG Fc could pull down more A64T-mutant Env protein (Fig 9E). Our analysis of Env protein and viral RNA levels revealed that the J1-A64T virus displayed enhanced adsorption to DF-1 cells, indicating the A64T mutation increases Env-receptor binding affinity (Fig 9F).

## Discussion

RNA viruses leverage error-prone replication to generate quasispecies through competitive selection (immune/antiviral pressures) and genetic drift, driving environmental adaptation and evolutionary optimization of viral fitness [31,32]. Decades of virus research have used targeted mutagenesis to study how mutations affect protein structure, function, and viral fitness. While this approach has revealed key insights into protein activity, replication, and pathogenicity, it is limited by its narrow focus on hypothesized critical mutations, often identified through homology. DMS enabled modeling natural selection's deterministic effects by tracking positive selection (enriching beneficial mutations) and negative selection (purging deleterious ones), making certain evolutionary pathways more probable. Comprehensive viral evolution prediction requires balancing persistent adaptation to stable environments with rapid responses to ecological changes [33].

Through high-throughput genetic mapping of ALV-J envelope mutations, we identified fitness effects that recapitulate natural evolutionary patterns while uncovering novel phenotypes. Serial passaging confirmed that viral fitness was restored through purifying selection, with N-terminal mutations (first 80 amino acids) subjected to particularly strong negative selection, indicating the functional importance of this region. Comparative structural analysis revealed distinct organization among retroviral Env proteins: while HIV-1 gp120 maintains conserved C1-C5 domains and a V3 tropism loop, MLV Env utilizes an N-terminal receptor-binding domain stabilized by proline-rich motifs. These findings suggest that systematic mapping of ALV-J Env's functional domains is crucial for understanding its viral features.

Although ALV-J Env contains previously annotated hypervariable (hr1/hr2) and variable (vr2/vr3) regions [12–16], our analysis of 562 viral strains indicates that mutation hotspots do not fully align with these classical domains. The primary reason for this discrepancy is that the current hr/vr classification system follows the framework originally defined for ALV subgroups A–E. Unlike ALV-A–E [34], natural mutations in ALV-J predominantly affect receptor binding affinity rather than altering cellular tropism. Consequently, there is a need to update the definition of variable regions based on the biological characteristics of ALV-J. Our results (Fig 5A) show that amino acid variations in ALV-J SU are mainly concentrated in the following five regions: 45–75, 110–120, 145–155, 185–220, and 238–245. Accordingly, we propose redefining these regions as vr1 through vr5.

Serial passaging revealed a minor viral subpopulation with in-frame Env indels. Notably, these mutations mirrored the positions and amino acid changes observed in natural ALV-J strains, consistent with previous reports of insertions in ALV-J quasispecies during avian infections [35]. These findings identify mutation-prone hotspots in ALV-J's genome, with indel conservation across wild strains suggesting functional significance. Their recurrent emergence in distinct lineages indicates adaptive potential. While most glycosylation mutants remained viable, N/C-terminal mutations reduced viral titers by >90%, reflecting their substantial fitness cost. The preservation of these glycosylation motifs across strains underscores their essential role. Additionally, ALV-J's TM protein showed no mutations with a frequency greater than 1%, with a significantly lower mutation rate than SU in natural isolates, indicating strong functional constraints.

Phylogenetic analysis of ALV-J strains revealed three temporal clusters corresponding to key epidemiological shifts. Early strains caused myelocytomas in broilers. The 2004 outbreak introduced hemangiomas in layers [18]. An eradication program brought layer infection under control within five years, but 2018 marked a resurgence, with myelocytomatosis reappearing in broilers and indigenous breeds nationwide in China [36]. Phylogenetic analysis confirmed that contemporary strains now form distinct evolutionary clades. DMS identifies critical amino acid mutations consistent with the evolutionary trajectory of natural isolates. Our findings revealed a strong correlation between fitness effects and the natural occurrence of mutations. These findings demonstrate dual significance: (1) A strong correlation between RC values and viral evolutionary pattern validates experimental reliability; (2) The identified positively-selected sites confer substantial replicative advantages critical for viral propagation. The original J1 strain closely matched the 2009–2012 isolates, while ‘evolved J1’ resembled the 2017–2019 strains (particularly TBC-J4/J6). Our prior studies showed the replicative advantage of TBC-J6 [37], suggesting it occupies a key adaptive niche, with its mutations likely representing ALV-J's future evolutionary direction.

We compared the correlation scatter plot of all RC values between the library virus (passage 10) and SPF chickens (30 days) in S3 Fig. The key mutation sites showed consistent patterns both in *vivo* and in *vitro*, and these findings were reproducible across three independent biological replicates. This indicates that positively-selected sites enhance viral propagation. In this study, we also used J1 strain as a reference virus to infect SPF chickens, monitoring the selection dynamics of wild isolate in *vivo*. At 30 days post-infection, the wild-type strain showed mutations (>1% frequency) at 23 sites, with 60.9% (14/23) matching in *vitro* selection results (S7 Fig). While mutation profiles largely overlapped, primary divergence occurred in the 190–220 region containing receptor-binding domains, suggesting potential links to cellular immunity. The in *vivo* patterns also resembled previous viral quasispecies data [38], supporting combined in *vivo*/*in vitro* approaches for predicting viral evolution.

In the animal experiment, both J1 and evolved J1 strains established persistent viremia in SPF chicken, with evolved J1 showing significantly higher viral shedding (from week 2) and greater organ viral loads. IHC revealed more ALV-J+ cells in evolved J1-infected tissues. We propose a strong correlation between viral replication levels in vivo and pathogenicity.

Ultimately, two critical sites were selected for functional validation: a putative N-linked glycosylation motif and a Furin protease cleavage site. Both mutations demonstrated significantly enhanced viral replication efficiency. Envelope glycosylation critically determines fitness through immune evasion and host adaptation, with N-linked glycosylation known to boost HIV-1 infectivity [39,40] and receptor-binding capacity of envelope proteins in influenza viruses [41] and SARS-CoV-2 [42]. In this study, the A64T mutation emerged as a dominant mutation site in both in *vivo* and in *vitro* screenings. Given its location in the receptor-binding domain, we consider it a critical site affecting viral adsorption. Additionally, the glycans may potentially mask known antigens. Notably, mutations at proteolytic cleavage sites exert profound virological implications. The multibasic cleavage motifs within the hemagglutinin (HA) glycoprotein critically drive influenza virulence [43]. Similarly, SARS-CoV-2 variants with altered Furin cleavage sites (P681R) showed increased transmission and pathogenicity [44]. We now demonstrate that ALV-J envelope cleavage site mutations boost proteolytic processing and replication kinetics, though the structural and evolutionary mechanisms require further investigation.

It is important to acknowledge certain methodological limitations in this study. The high-throughput sequencing approach and the associated statistical analysis of replication capacity (RC) values may fail to fully capture synergistic interactions among multiple mutations. This methodological framework inherently constrains the ability to assess the combined phenotypic effects of mutations that are adjacent or clustered in the sequence. However, a major strength of this work is its potential to model viral adaptation quantitatively. These models can be used to monitor and anticipate the emergence of new variants.

## Materials and methods

### Ethics statement

The animal experiments were conducted in accordance with protocols approved by the Institutional Animal Care and Use Committee of Yangzhou University (Approval No. 202402064; Date: 26 February 2024).

### Cells and viruses

DF-1 cells were maintained in Dulbecco’s modified Eagle’s medium (DMEM) supplemented with 10% fetal bovine serum (FBS). ALV-J strain J1 (PV523728) was kept in our laboratory.

### Determination of viral titer

Viral titers in cell culture supernatants were determined by TCID₅₀ using the Reed-Muench method. Serial logarithmic dilutions (10 ⁻ ¹ to 10 ⁻ ⁸) of each sample were inoculated into eight replicate wells per dilution. After 7 days of incubation, viral replication was assessed by ELISA detection of the capsid protein p27.

### Generation of random mutation library of ALV-J Env

The ALV-J Env mutant library was generated using error-prone PCR with Mutazyme II polymerase (Strategene, USA) on the pMD-J1 infectious clone template. Following PCR amplification of the SU fragment, we purified and recombined it with the pMD-J1 backbone using Exnase II (Vazyme, China) [17]. The resulting full-length proviral genomes were electroporated into SS320 competent cells and plated on ampicillin-containing LB agar.

### Generation and passaging of mutant viruses

The ALV-J mutant library was generated by transfecting DF-1 cells with 2.5 μg plasmid DNA using TransIT-LT1 (Mirus, USA), supplemented with 2 μg/mL polybrene (Beyotime, China) to enhance efficiency. Wild-type ALV-J transfection was performed in parallel. Supernatants were harvested at 3 days post-transfection and stored at -80 °C.

For infection studies, DF-1 cells were inoculated with mutant or wild-type virus (MOI = 0.1). After 2h adsorption, medium was replaced with DMEM containing 1% FBS. Viral replication was assessed by p27 levels at 1, 3, and 5 dpi. At 5 dpi, cells underwent freeze-thaw cycles followed by centrifugation - half for subsequent passages (10 in total) and half for RNA extraction (TRIzol, ThermoFisher). Viral RNA was collected at generations 1, 4, 7, and 10, with polybrene (2 μg/mL) added during each passage.

### Sanger sequencing and Illumina deep sequencing

RNA samples were reverse-transcribed into cDNA using HiScript III first strand cDNA synthesis kit (Vazyme, China). Four primer pairs were used to amplify the SU gene, while three primer pairs amplified the TM gene, using high-fidelity DNA polymerase (Vazyme, China) under standard cycling conditions (95 °C 1min, followed by 40 cycles of 95 °C 20s/53 °C 20s/72 °C 30s, and final extension at 72 °C for 1 min).

PCR products were verified by 3% agarose gel electrophoresis. After purification and normalization to 30 ng/μL, 800 ng of each sample was used for library preparation. The process included end repair, phosphorylation, dA-tailing, and adapter ligation, followed by purification with DNA Clean Beads. A second PCR added flowcell-binding sequences and indices for multiplexing. Final libraries were sequenced on Illumina HiSeq 2500, generating >8 million 150 bp paired-end reads per sample.

For quality control, raw reads with less than 90% Q30 bases were filtered out using NGS_QC Toolkit (v2.3.3). Filtered reads were downsampled to 90,000 paired-end reads (150 bp), providing ~100,000 × coverage. Reads were mapped to the reference genome using BWA, with unmapped reads removed using Samtools. Alignment results were visualized using IGV 2.19.1.

The complete *env* gene was amplified and cloned into pUC19 vector (Takara Bio) for Sanger sequencing validation. All primer sequences are provided in S4 Table.

### Analysis of deep-sequencing data

Deep sequencing accuracy was influenced by multiple factors, including reagent-induced mutations from the HiScript III cDNA kit (Vazyme, China) (1.4 × 10 ⁻ ⁶/site) and high-fidelity enzyme (Vazyme, China) (3 × 10 ⁻ ⁵/site), as well as ALV's spontaneous mutation rate (4.6 × 10 ⁻ ⁵/site/generation). To ensure data reliability, we performed experiments in triplicate and implemented a stringent frequency cutoff (＜3 × 10 ⁻ ⁴) to filter out low-frequency artifacts and improve signal-to-noise ratio.

We established a Replication Capacity (RC) metric to assess mutation fitness effects through single-site saturation mutagenesis. This approach isolates individual mutation impacts while minimizing epistatic interference from linked mutations. The high-density coverage enables robust RC calculations by averaging out confounding epistatic effects, as previously validated [26].

The mutation frequency at each specific site is calculated as the count of a particular base at that position divided by the total number of bases sequenced for that position.


$$\text{RC value }mutant\;i=\frac{\text{ mutation frequency of mutant }i\;(\text{infection})\;}{\text{mutation frequency of mutant }i\;(\text{plasmid})}$$

### Screening key mutation sites on the envelope protein

After filtering out low-confidence data, we calculated the RC value for each mutation site across all samples. The mutations were then categorized into four groups based on their RC values: level A (RC > 1.5), level B (0.5 ≤ RC ≤ 1.5), level C (0.05 ≤ RC < 0.5) and level D (RC < 0.05).

At the molecular level, each nucleotide can mutate to any of three alternative bases, so every triplet codon has nine possible single-nucleotide variants, potentially leading to different amino acid substitutions. Different amino acid changes often produced varying RC values. Replicates with inconsistent RC values were labeled ‘uncertain’ and excluded from further analysis.

We established quantitative criteria to systematically evaluate envelope protein mutations, termed “amino acid mutation fitness values (AA mutation fitness values).

- Variable mutations: A > 0
- Tolerated mutations: A = 0 AND (A-C-D)/(A + B + C + D) ≥ -0.5
- Non-tolerated mutations: A = 0 AND (A-C-D)/(A + B + C + D)＜-0.5

### Alignments and phylogenetic analyses of envelope sequences

To analyze their evolutionary origins, maximum likelihood (ML) trees were constructed using MEGA X (version 10.2.6; https://www.megasoftware.net) with 1,000 bootstrap replicates for reliability assessment. Reference ALV-J SU sequences were obtained from GenBank (S1 Table).

### Construction of infectious clones of ALV-J and protein expression vectors

We generated ALV-J infectious clones containing specific mutations via site-directed mutagenesis [17,45], with all modifications verified by Sanger sequencing. We then constructed Env protein expression vectors carrying the A64T and H304R mutations by cloning the Env open reading frame (ORF) into the pCAGGS vector [17].

### Detection of viral adsorption through RT-qPCR

A previously described probe-based multiple qPCR method [46] was used to detect ALV-J genomic RNA in the cell supernatant (copies/mL) and lysates (copies/μg). The viral genomic RNA copy numbers were calculated using a known copy number standard curve.

To evaluate virus adsorption efficiency, DF-1 cells precooled at 4 °C were incubated with wild-type or mutant ALV-J containing equal viral genomic RNA (10^7^ copies/mL) for 2 h at 4 °C, which allowed virus binding but not internalization. Unbound ALV-J particles were washed three times with ice-cold PBS. Then viral and cell RNA were extracted as described above.

### Detection of proviral loads through qPCR

Genomic DNA was extracted using the FastPure Blood/Cell/Tissue/Bacteria DNA Isolation Mini Kit (Vazyme, Nanjing, China) following the manufacturer’s instructions. The proviral genome load in the chicken genome was quantified as previously described [47], using the single-copy housekeeping gene OVO as a reference [48].

### Western blot, ELISA and Immunofluorescence assay

Western blotting was performed as previously described [49] using anti-ALV-J Env mAb JE9, anti-p27 mAb 5D3 (kept in our lab), anti-gp37 mAb 3B5 (kept in our lab), anti-GAPDH mAb (Abcam, UK), HRP-labeled anti-mouse IgG (H + L) antibodies (Jackson, USA) and HRP-labeled anti-rabbit IgG (H + L) antibodies (Jackson, USA). The mAb 5D3 [50,51] specifically recognizes the ALV capsid protein p27, while mAb JE9 targets both the SU protein and its precursor Env of ALV-J [52]. Additionally, mAb 3B5 binds to the TM protein and precursor Env of ALV-J.

A sandwich ELISA assay was performed to detect the capsid protein p27 in the samples [53]. For DF-1 cultures, 100 μL of freeze-thawed supernatant was analyzed. Cloacal swabs were suspended in 500 μL PBS, freeze-thawed twice, with 100 μL lysate tested. For blood samples, 60 μL plasma was incubated with DF-1 cells for 7 days before testing 100 μL of processed supernatant. All assays were performed with a final positive threshold set at OD_650_ = 0.1.

Using our previously developed ELISA methods, we tested chicken serum for antibodies against ALV-J Env [52] and capsid protein p27 [54], with an OD_450_ cut-off value of 0.1.

Immunofluorescence assay was performed as previously described [55] using anti-ALV-J Env mAb JE9, anti-p27 mAb 5D3 and FITC-labeled goat anti-rabbit IgG (H + L) antibodies (Jackson, USA).

### Co-Immunoprecipitation

Co-Immunoprecipitation (Co-IP) was performed as previously described [17]. Briefly, DF-1 cells were transfected with plasmids encoding mutant Env proteins or pCAGGS-NHE1-ECL1-rIgG (expressing ALV-J receptor NHE1's first extracellular loop fused to rabbit IgG Fc). After 48 hours, cells were lysed and incubated with Protein A/G Agarose beads (Santa Cruz Biotechnology, USA) at 4°C overnight. The immunoprecipitates were then eluted by boiling in SDS-loading buffer for western blot analysis.

### Purification of fusion protein and LC-MS analysis

DF-1 cells were transfected with pCAGGS-SU-rIgG (ALV-J SU fused to rabbit IgG Fc). After 3 days of culture, the supernatant was collected and concentrated to harvest the fusion protein using HiTrap Protein G HP (Cytiva, Uppsala, Sweden) following a published protocol [17].

The SU-rIgG glycoprotein (100 µg) was reduced in 50 mmol/L NH₄HCO₃ buffer. 10 µL samples were analyzed by SDS-PAGE. Gel slices were excised, destained with ammonium bicarbonate/acetonitrile (1:1, v/v), and incubated in acetonitrile. After rehydration in DTT/ammonium bicarbonate, slices were alkylated at 56°C for 1 h, then digested with chymotrypsin (Promega, USA) at 30°C for 16 h. Peptides were extracted with 5% TFA-50% ACN-45% ddH₂O at 37°C for 1 h, centrifuged, and the supernatant collected for nanoLC-MS/MS analysis.

Chromatographic separation was performed on an EASY-nLC 1200 system using mobile phases A (0.1% formic acid in water) and B (0.1% formic acid in 80% acetonitrile). Raw data were processed with Byonic software (v4.2.4) for identification and quantitative analysis.

### Animal experiments

Three 1-day-old SPF White Leghorns were inoculated with 5,000 TCID₅₀ ALV-J library virus via subcutaneous injection, while three additional chickens served as negative controls (n = 3, 3 males). Cloacal swabs were collected weekly for 30 days before euthanasia. Serum, peripheral blood lymphocytes, and tissues were collected for ELISA, RNA-seq and histopathology, respectively.

Thirty 1-day-old SPF chickens were randomly divided into three groups (n = 10, with 5 males and 5 females each) receiving either the J1 strain, evolved J1 strain (both at 10,000 TCID₅₀), or diluent control via subcutaneous injection. Weekly cloacal swabs and blood samples were collected for four weeks, followed by euthanasia at 30 days with collection of multiple organs (liver, heart, spleen and kidney) for histopathology, DNA extraction, and viral load quantification by qPCR.

### Histopathological examination and immunohistochemistry

Tissues were analyzed by histopathology. Briefly, liver and spleen samples were fixed in 10% formalin, alcohol-dehydrated, and paraffin-embedded for H&E staining.

The immunohistochemistry (IHC) procedure was performed on paraffin-embedded tissue sections. After deparaffinization and antigen retrieval, endogenous peroxidase activity was blocked. Sections were then incubated with anti-p27 mAb 5D3(1ng/μL) overnight at 4°C, followed by HRP-conjugated secondary antibody. DAB was used as the chromogen, and slides were counterstained with hematoxylin. Finally, sections were dehydrated, cleared, and mounted for microscopic examination.

### Structure analyses and data processing

The trimer of the ALV-J Env protein was modeled using AlphaFold2.3.2 [56]. Molecular graphics and analyses were performed using the PyMol 3.1 software.

Statistical analysis was performed using the GraphPad Prism 5 software (GraphPad Software, Inc.). Statistical significance was set at P < 0.05.

## Supporting information

S1 Table The ALV-J strains in this study. (XLSX)

S2 Table DMS identifies deletion/insertion mutations that closely match those found in naturally occurring ALV-J strains. (DOCX)

S3 Table RC values of N/O-glycosylation sites in ALV-J mutation library. (DOCX)

S4 Table Primers used in this study. (DOCX)

S1 Fig Replication kinetics of the library virus and the ALV-J J1 strain over ten serial passages. (TIF)

S2 Fig Detection of antibodies against the envelope protein Env and capsid protein p27 in chicken serum. Antibodies against ALV-J SU (A) and p27 (B) were detected in chickens on days 15 and 30.
(TIF)

S3 Fig Functional Envs and yields measurements that are well correlated among replicates. Correlations between 3 replicates in the mutant library group at each generation in each site in envelope for all mutations.
(TIF)

S4 Fig Distribution of RC values for all mutations across different samples. (TIF)

S5 Fig Sanger sequencing analysis of Env gene mutations in G10-passaged viruses. (TIF)

S6 Fig Detection of N-glycosylation at the N62 site of the ALV-J SU protein expressed in eukaryotes by LC-MS. (A) Treatment of the eukaryotic ALV-J SU protein (with a rabbit IgG Fc tag) with PNGase F, which cleaves N-linked glycans, reduced its molecular weight from approximately 100 kDa to 55 kDa. (B) LC-MS identified a peptide containing an N62 glycosylation modification. (C) N62 is occupied by high-mannose–type glycans containing five mannose and two N-acetylgalactosamine (GalNAc) residues.
(TIF)

S7 Fig Per-codon frequency of ALV-J J1 in chickens at 30 days. (TIF)
