# Supplementary material for: Mapping the mutational landscape of an avian retrovirus envelope protein across its evolutionary trajectory
Source: PLoS Pathog. 2026 Mar 31;22(3):e1014110. doi: 10.1371/journal.ppat.1014110 (PMC13048475; doi:10.1371/journal.ppat.1014110)
Supplement: S2 Table — (DOCX) [file ppat.1014110.s002.docx]

**S2 Table Deep mutational scanning identifies deletion/insertion mutations that closely match those found in naturally occurring ALV-J strains**

| **Deletion/insertion mutation** | **Matched nature ALV-J isolates** |
| --- | --- |
| △K113 | JS09GY3 (GU982308), HUB09WH02 (HQ634804), SDAU1102 (KU159178) |
| △N216-G219 | TBC-J6 (MT409625), UD-J1 (AF305091), GD19ZH02 (MT538249) |
| △S214 | HUE2023 (PQ063994), GZ2024GY02J (PV239801) |
| N117-S118▽SR | JS14XJO3 (MT783254), GD14J2 (KU500032) |
| N117-S118▽NN | GD19ZH02 (MT538249), GD16FS01 (MT538237) |
| N239-S240▽Q | HLJ10SH04 (HQ634814), 4817 (AF247385) |
| N239-S240▽GN | JS16JH10 (MG700542), GD0510A (EF103132) |
| N239-S240▽K | JGD18QY22 (MN262620), JS18YZ01 (MN735303) |
