# Supplementary material for: Mapping the mutational landscape of an avian retrovirus envelope protein across its evolutionary trajectory
Source: PLoS Pathog. 2026 Mar 31;22(3):e1014110. doi: 10.1371/journal.ppat.1014110 (PMC13048475; doi:10.1371/journal.ppat.1014110)
Supplement: S3 Table — (DOCX) [file ppat.1014110.s003.docx]

**S3 Table RC values of N/O-lined glycosylation sites in ALV-J mutation library (Generation 1 to Generation 10)**

N-linked glycosylation sites in natural isolates were included if their positions aligned within ±2 amino acids of the corresponding sites in the reference strain J1, accounting for potential insertion-deletion mutations

| **Gylcan type** | **Position** | **RC values**  **Generation 1** | **RC values**  **Generation 4** | **RC values**  **Generation 7** | **RC values**  **Generation 10** |
| --- | --- | --- | --- | --- | --- |
| N-glycan | N17/T19 | 0.27 | 0.21 | 0.16 | 0.07 |
| O-glycan | T32 | 0.42 | 0.22 | 0.06 | 0.09 |
| N-glycan | N56/T58 | 0.26 | 0.14 | 0.26 | 0.19 |
| N-glycan | N78/T80 | 0.74 | 0.4 | 0.45 | 0.29 |
| N-glycan | N98/T100 | 0.81 | 0.39 | 0.19 | 0.11 |
| N-glycan | N101/T103 | 4.21 | 2.09 | 0.76 | 0.57 |
| N-glycan | N116/S118 | 0.72 | 0.35 | 0.12 | 0.09 |
| N-glycan | N129/T131 | 0.65 | 0.3 | 0.07 | 0.03 |
| N-glycan | N157/S159 | NA | NA | NA | NA |
| N-glycan | N183/T185 | 1.28 | 0.85 | 0.24 | 0.16 |
| N-glycan | N191/T193 | 1.40 | 0.93 | 0.12 | 0.11 |
| N-glycan | N209/T211 | 0.65 | 0.43 | 0.03 | 0.02 |
| N-glycan | N216/T218 | 0.96 | 0.74 | 0.06 | 0.05 |
| N-glycan | N223/T225 | 0.86 | 0.59 | 0.02 | 0.02 |
| N-glycan | N239/T241 | 1.73 | 1.08 | 0.11 | 0.09 |
| N-glycan | N247/S249 | 1.73 | 0.98 | 0.1 | 0.06 |
| O-glycan | T284 | 0.52 | 0.3 | 0.14 | 0.05 |
| N-glycan | N289/T291 | 0.34 | 0.21 | 0.08 | 0.07 |
| N-glycan | N299/T301 | 0.84 | 0.52 | 0.15 | 0.16 |
