# Supplementary material for: Mapping the mutational landscape of an avian retrovirus envelope protein across its evolutionary trajectory
Source: PLoS Pathog. 2026 Mar 31;22(3):e1014110. doi: 10.1371/journal.ppat.1014110 (PMC13048475; doi:10.1371/journal.ppat.1014110)
Supplement: S4 Table — (DOCX) [file ppat.1014110.s004.docx]

**S4 Table Primers used in this study**

| **Primer** | **Sequence** | **Function** |
| --- | --- | --- |
| PE150-1-F | TTCTGTTGTCCCAGGGGTGG | SU gene amplification for Next Generation Sequencing |
| PE150-1-R | CAAGGGAGGGTTGTATTTAGAG |  |
| PE150-2-F | GATACAGGCTCTAAATACAAC | SU gene amplification for Next Generation Sequencing |
| PE150-2-R | ACCATCGTCCATGATTGGTTG |  |
| PE150-3-F | TATGTCAACCAATCATGGACG | SU gene amplification for Next Generation Sequencing |
| PE150-3-R | CAATACCTCTGTCTTATTCTC |  |
| PE150-4-F | GGAATGGAATTATTATGCATATG | SU gene amplification for Next Generation Sequencing |
| PE150-4-R | CAGACGAGAGATGACTCATCGAG |  |
| PE150-5-F | ATGGGCCGAACATTACGGGT | TM gene amplification for Next Generation Sequencing |
| PE150-5-R | GCGTGCCGGATACTGACGTTAATCCT |  |
| PE150-6-F | ACATCATTAATATTGAATGCG | TM gene amplification for Next Generation Sequencing |
| PE150-6-R | CAAACGTGCGCGTAAACCAATC |  |
| PE150-7-F | GAAGATACGGGTGGAAGATGATC | TM gene amplification for Next Generation Sequencing |
| PE150-7-R | CACATCGCGCTTGGGAACCACAG |  |
| ALV-J Env-F | GAGGTGACTAAGAAAGATGAGGCGAGCC | Env gene amplification for Sanger Sequencing |
| ALV-J Env-R | CCATCAACCCAGGTGCACACCAATG |  |
| ALV-J-NF | TTGCAGGCATTTCTGACTGG | Detection of ALV-J proviral loads |
| ALV-J-NR | ACACGTTTCCTGGTTGTTGC |  |
| ALV-J probe | FAM-CCTGGGAAGGTGAGCAAGAAGGA-BHQ1 |  |
| OVO-F | CACTGCCACTGGGCTCTGC | Genomic housekeeping gene |
| OVO-F | GCAATGGCAATAAACCTCCAA |  |
| OVO-probe | ROX-AGTCTGGAGAAGTCTGTGCAGCCTCCA-BHQ2 |  |
